# Retrograde EUS-guided ileocolostomy for malignant small-bowel obstruction

**DOI:** 10.1016/j.vgie.2023.12.004

**Published:** 2023-12-13

**Authors:** Michael Lajin, Carl Eric Orr, Fateh Bazerbachi

**Affiliations:** 1 Sharp HealthCare, San Diego, California; 2 CentraCare, St. Cloud, Minnesota

## Introduction

Luminal and biliary obstructions are common challenges in patients with advanced GI malignancies. This subset of patients is more prone to adverse events after surgery owing to poor nutritional and functional status. Surgical interventions are also associated with longer recovery and interruption of cancer treatments. EUS-guided biliary and luminal interventions are being increasingly used in these settings.^1^ Various EUS-guided transmural techniques such as gastroenterostomy,^2^ enterocolostomy,^3^ decompression of afferent loop obstruction,^4^ and biliary^5^ and gallbladder drainages have been reported with good clinical outcomes despite challenging anatomies. EUS-guided interventions offer faster recovery and the ability to resume diet and cancer treatment sooner than surgery.^6^ Compared with percutaneous interventions, EUS-guided endoscopic interventions offer patients a better quality of life by allowing them to resume a regular diet and avoiding percutaneous tubes and drainages in addition to requiring fewer interventions.^7^

## Case

**Figure 1:**
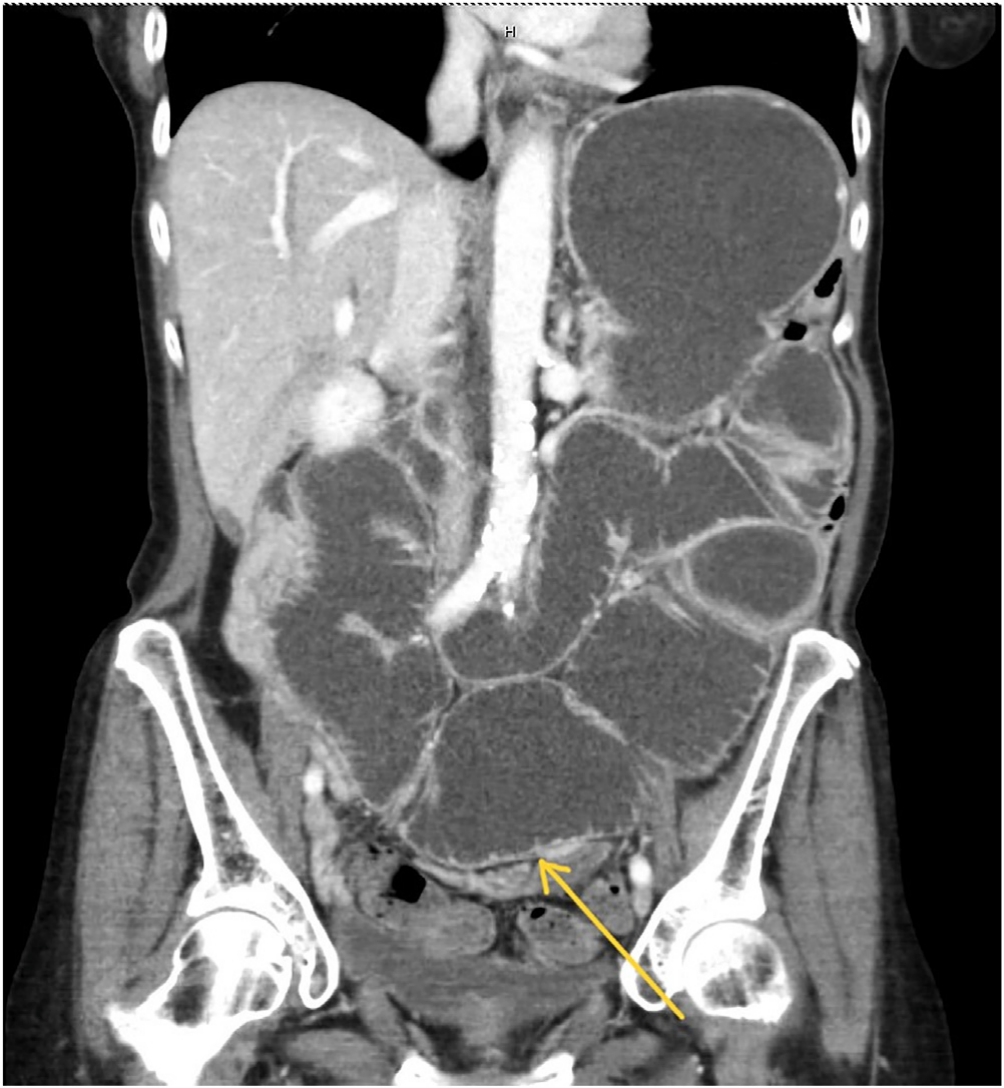
Abdominal/pelvic CT image demonstrating small-bowel obstruction. The *yellow arrow* points to the opposition between the ileum and the rectosigmoid colon.

We present the case of a 64-year-old woman with a history of pulmonary adenocarcinoma and a history of perforated goblet cell carcinoid tumor of the appendix. She underwent extensive cytoreductive surgery with heated intraperitoneal chemotherapy 5 years prior. She had a recent small-bowel obstruction requiring exploratory laparotomy with extensive lysis of adhesions and resection of recurrent tumor. Four months later, she presented with abdominal distension, nausea, and vomiting. A CT scan showed evidence of distal small-bowel obstruction. A nasogastric tube was placed for decompression. She was considered a poor candidate for further surgical interventions. After reviewing her CT scan, we noted good opposition between the rectum and a distended distal ileal loop (Fig. 1). After a multidisciplinary discussion, a decision was made to proceed with EUS-guided ileocolostomy using a lumen-apposing metal stent.

### Procedure

**Figure 2:**
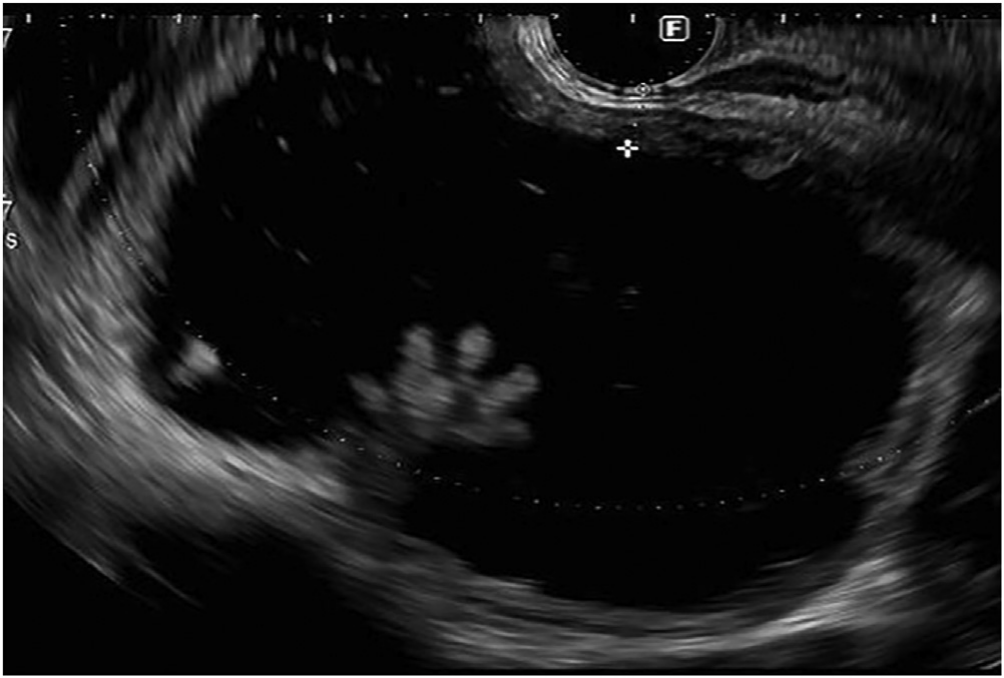
EUS image of a distended ileal loop opposed to the rectosigmoid colon.

**Figure 3:**
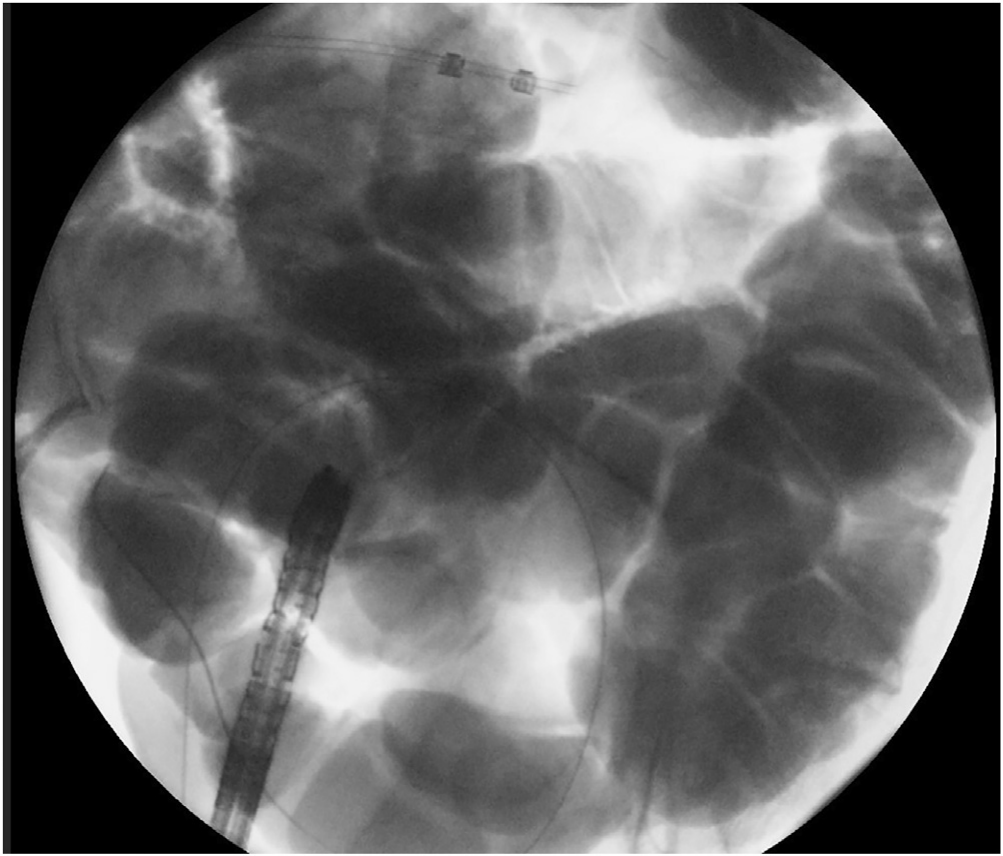
Fluoroscopic image of a distended ileal loop opposed to the rectosigmoid colon.

**Figure 4:**
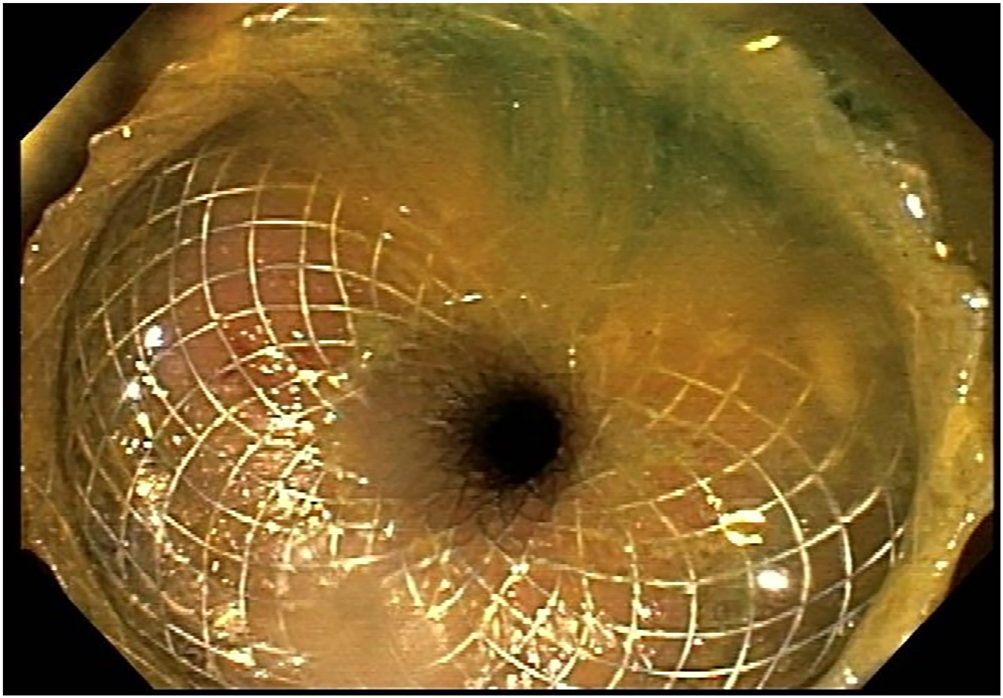
Endoscopic image after full deployment of the lumen-apposing metal stent.

**Figure 5:**
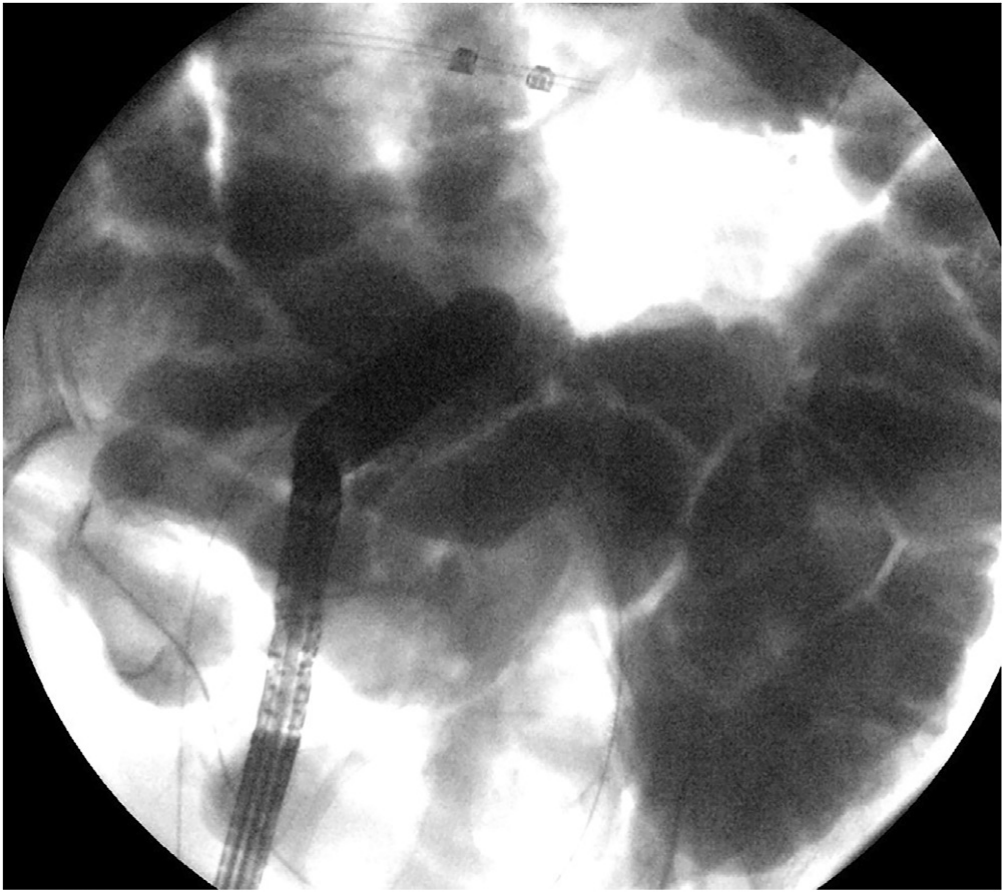
Fluoroscopic image of balloon dilation of the lumen-apposing metal stent.

**Figure 6:**
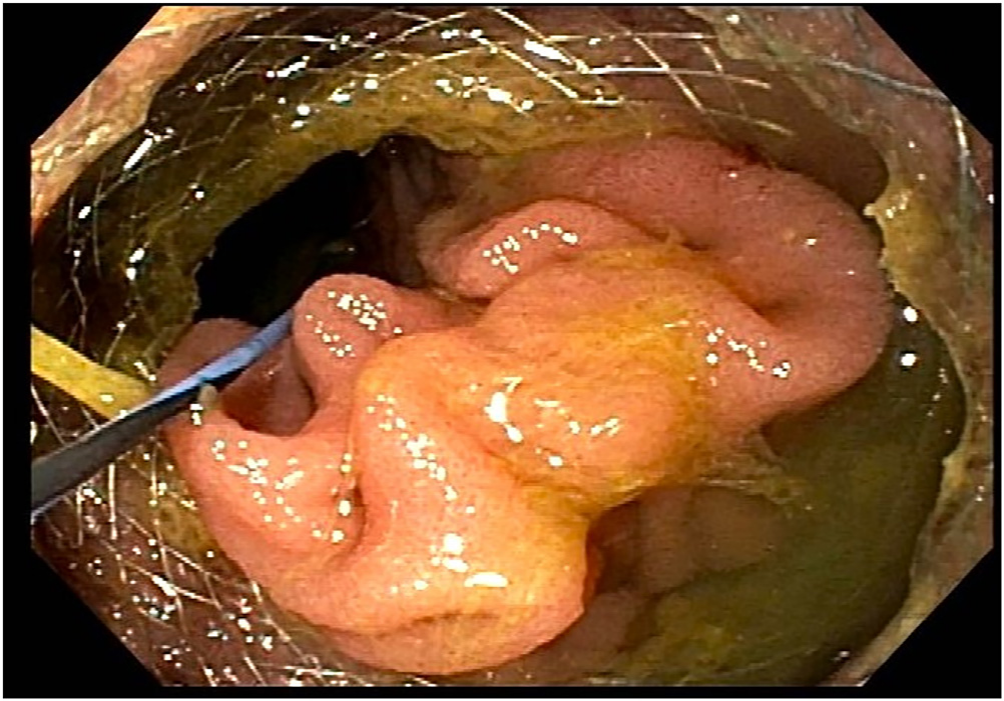
Endoscopic image of the ileocolostomy after dilating the lumen-apposing metal stent.

The patient received a prophylactic dose of antibiotics. The procedure was done with the patient under general anesthesia. The patient could not receive bowel preparation owing to complete obstruction. An endoscope was advanced to the sigmoid colon. A wire was left in the colon as the endoscope was removed. Contrast with methylene blue was injected through the nasogastric tube to distend the distal ileal loops. A linear echoendoscope was advanced to the sigmoid colon, guided by the wire and then withdrawn slowly. At the rectosigmoid junction, a distended ileal loop was identified on EUS (Fig. 2) and fluoroscopy (Fig. 3). This loop was opposed to the colon with no intervening organs or blood vessels. An electrocautery-enhanced lumen-apposing metal stent (15 × 10 mm) was deployed, connecting the colon to the ileum proximal to the obstruction (Fig. 4). Methylene blue was seen flowing from the stent after deployment. The stent was dilated to 15 mm (Figs. 5 and 6).

### Outcome

**Figure 7:**
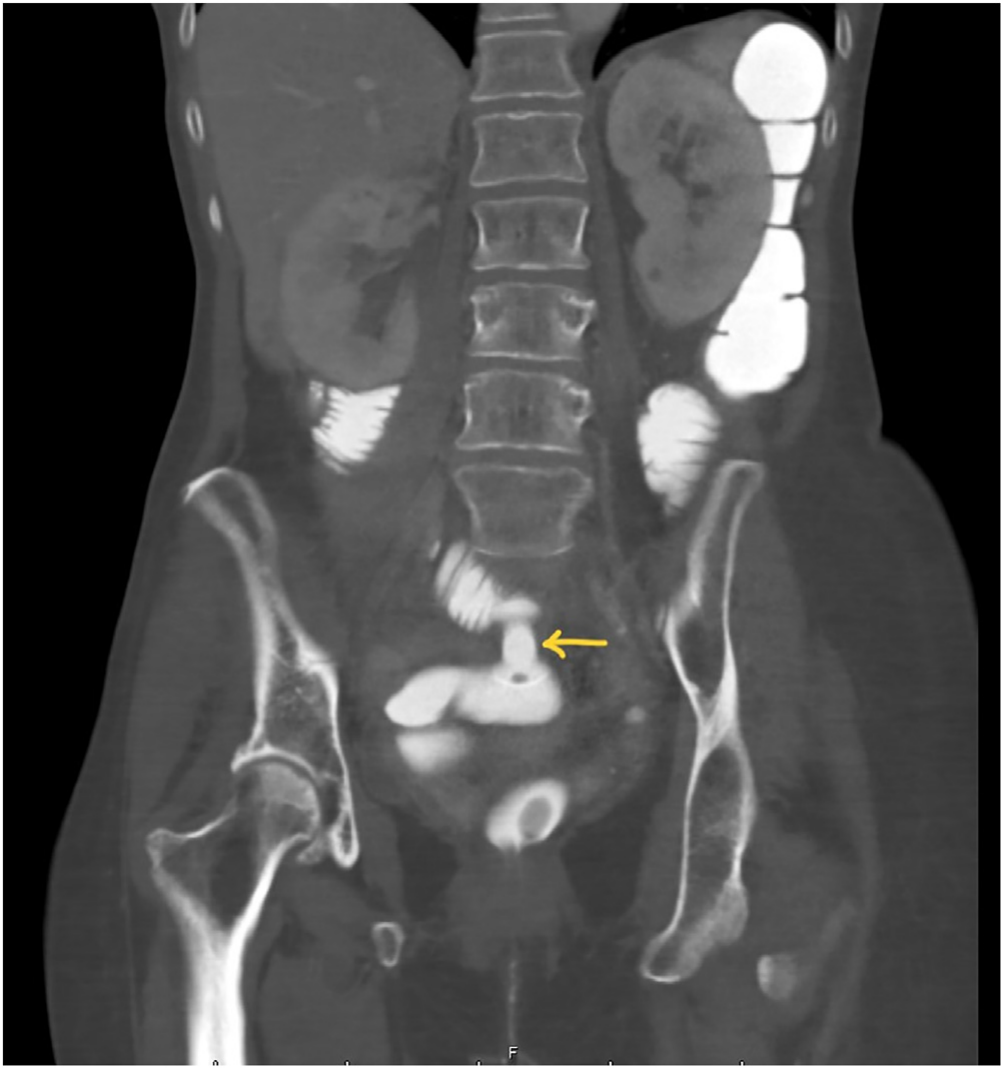
Coronal CT image of the ileocolostomy (*yellow arrow*) and decompression of the small bowel.

**Figure 8:**
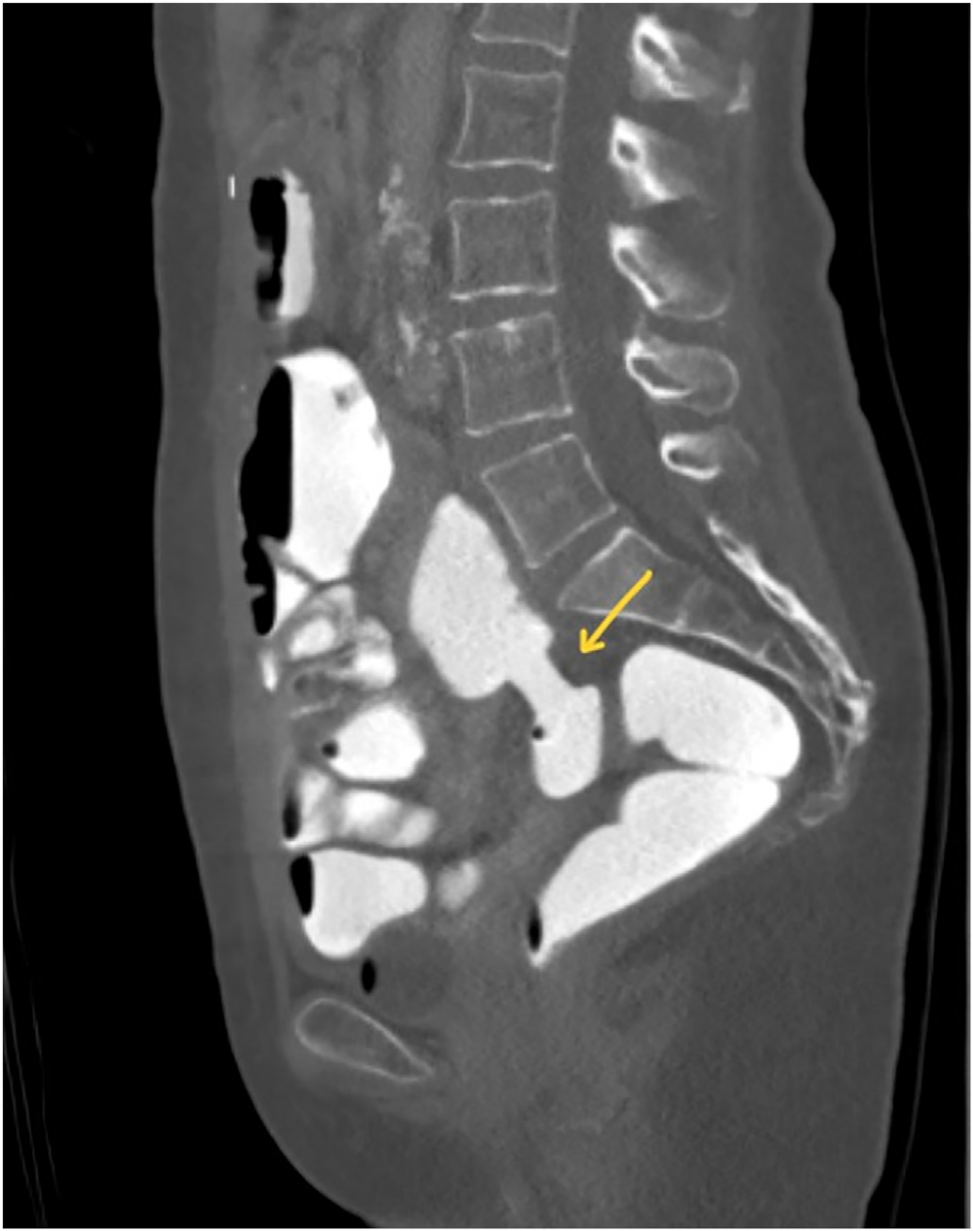
Sagittal CT image of the ileocolostomy (*yellow arrow*) and decompression of the small bowel.

There were no adverse events after the procedure. Postoperative CT confirmed a successful ileocolostomy with complete decompression of the small bowel (Figs. 7 and 8). Her diet was advanced to a soft diet, and she was discharged home under hospice care. She had no recurrence of small-bowel obstruction until she died 3 months later.

## Conclusion

EUS-guided interventions to treat luminal and biliary obstructions in patients with advanced GI malignancies offer faster recovery and a better quality of life compared with surgical and percutaneous interventions, respectively. Retrograde EUS-guided ileocolostomy appears to be an effective palliative tool in select patients with advanced malignancies presenting with small-bowel obstruction. Additional studies are required to determine the safety and effectiveness of this technique (Video 1, available online at www.videogie.org).

## Disclosure

The authors disclosed no financial relationships relevant to this publication.

## Supplementary data

Video 1 Retrograde EUS-guided ileocolostomy performed to palliate a malignant distal small-bowel obstruction.
